# Methanogens Within a High Salinity Oil Reservoir From the Gulf of Mexico

**DOI:** 10.3389/fmicb.2020.570714

**Published:** 2020-09-18

**Authors:** Glenn D. Christman, Rosa I. León-Zayas, Zarath M. Summers, Jennifer F. Biddle

**Affiliations:** ^1^School of Marine Science and Policy, University of Delaware, Lewes, DE, United States; ^2^Department of Biology, Willamette University, Salem, OR, United States; ^3^ExxonMobil Research & Engineering Company, Annandale, NJ, United States

**Keywords:** methanogen, methanol, oil reservoir, Archaea, metagenomics

## Abstract

Oil reservoirs contain microbial populations that are both autochthonously and allochthonously introduced by industrial development. These microbial populations are greatly influenced by external factors including, but not limited to, salinity and temperature. In this study, we used metagenomics to examine the microbial populations within five wells of the same hydrocarbon reservoir system in the Gulf of Mexico. These elevated salinity (149–181 ppt salinity, 4–5× salinity of seawater) reservoirs have limited taxonomic and functional microbial diversity dominated by methanogens, *Halanaerobium* and other Firmicutes lineages, and contained less abundant lineages such as Deltaproteobacteria. Metagenome assembled genomes (MAGs) were generated and analyzed from the various wells. Methanogen MAGs were closely related to *Methanohalophilus euhalobius*, a known methylotrophic methanogen from a high salinity oil environment. Based on metabolic reconstruction of genomes, the *Halanaerobium* perform glycine betaine fermentation, potentially produced by the methanogens. Industrial introduction of methanol to prevent methane hydrate formation to this environment is likely to be consumed by these methanogens. As such, this subsurface oil population may represent influences from industrial processes.

## Introduction

Methanogens have been noted in numerous anoxic, elevated salinity environments including deep sea brines, hypersaline microbial mats and soda lakes (McGenity, 2010). Noted in many of these environments are methylotrophic methanogens, which are expected to outcompete acetoclastic or hydrogenotrophic methanogens due to their use of methylated, non-competitive substrates (Sorokin et al., 2017). A recent study of produced fluids from unconventional shale reservoirs, in which salinity increased during continued production, showed that methylotrophic methanogens and halotolerant bacteria are likely linked through the fermentation of glycine betaine (Daly et al., 2016). In this environment, the production of trimethylamine occurred during glycine betaine fermentation in a *Halanaerobium* species, which then likely fueled the growth of *Methanohalophilus*, a halophilic and methylotrophic methanogen. These interactions are likely aided by the salt tolerance of the organisms.

Other high salinity environments include conventional crude oil reservoirs, which are accessible subsurface environments heavily influenced by industrial practices (e.g., drilling, production, and in some cases injected fluids from the surface). Microbial communities consisting of Archaea and Bacteria have been observed in reservoirs across wide salinities and temperatures (Pannekens et al., 2019). However, the provenance of community members is not always known. The communities observed are almost certainly a mix of both native organisms, anthropogenically introduced organisms, and organisms that are enriched due to industrial injections (Vigneron et al., 2017). Methanogens are consistently identified as members of these crude oil-associated communities (Pannekens et al., 2019).

Numerous methanogens have been isolated from oil wells (Belyaev et al., 1991). These methanogens include representatives from many metabolic groups including the hydrogenotroph *Methanocalculus halotolerans* (Ollivier et al., 1998) and methylotroph, *Methanohalophilus euhalobius* (Obraztsova et al., 1988; Davidova et al., 1997), both species isolated initially from high salinity oil reservoirs. These culture-dependent studies often concluded that methanogen diversity in oil fields may be limited to one or a few species. The impacts of temperature and salinity on methanogenic substrate usage and competition are still relatively poorly understood (Sorokin et al., 2017). More recent surveys of oil fields have employed culture-independent metagenomic approaches, during which the entire community is able to be captured and their metabolic preferences determined (Kotlar et al., 2011; Hu et al., 2016; Vigneron et al., 2017). In these studies, a restricted diversity of methanogens is still seen in individual wells, with only one or a few lineages of methanogens being present.

We hypothesized that the high salinity crude oil reservoirs in the Gulf of Mexico would be home to interesting microbes due to the selective pressures of salt. In this study, we examined multiple wells of a high salinity crude oil reservoir in the Gulf of Mexico via metagenomic analysis. We find what we interpret as a low biomass system, due to low DNA recovery. Our results suggest that microbial diversity within the system is limited. A dominant methanogenic lineage exists in this reservoir, and we hypothesize that metabolic interdependencies and industrial amendments may be what fuels the methanogens in this system.

## Materials and Methods

### Sample Site

Produced water samples were collected from production wells in the Hoover Field in the Hoover-Diana mini-basin located at the intersection of the Alaminos Canyon and East Breaks area of the western United States Gulf of Mexico. These wells are at 1400–1500 m water depth and the Hoover reservoir has a reported temperature of approximately 68°C (Thiagarajan et al., 2020). Oil and oil-solution gas are hosted in Plio-Pleistocene age reservoirs, and have been previously reported to be sourced from a Tertiary marine source interval (Hood et al., 2002). This system contains numerous salt structures and has been under production since 2000. All sampled wells produce from the same unit and were sampled at individual well heads. Oil in this system is regarded as partially biodegraded. Fluids within these reservoirs include saline brines with up to 5× seawater salinity, HA2 155 ppt; HA3 181 ppt; HA5 NA; HA6 149 ppt; MD1 168 ppt. Liquid hydrocarbon API gravity was also similar across all samples: HA2 25; HA3 26; HA5 25; HA6 25; MD1 25. Hydrate formation can be a costly issue for deep water wells and, as a standard practice in deep water oil and gas production, methanol is injected to try to prevent hydrate formation (Anderson and Prausnitz, 1986; Robinson and Ng, 1986).

Produced fluids for each well were flushed through a test separator for 30 min before samples were collected. Samples were allowed to briefly phase separate, and water phase was passed through 0.2 μm Sterivex filters until the filter was clogged, mostly by presence of liquid hydrocarbon droplets that remained in the sample. Filter cartridges were frozen immediately and stored at −80°C until processing. The total volume processed for each sample was: HA2 600 ml; HA3 250 ml; HA5 750 ml; HA6 550 ml (combination of 2 filters); MD1 685 ml (combination of 2 filters).

### DNA Extraction and Sequencing

DNA was extracted using a modified version of the Qiagen PowerWater Sterivex filter extraction kit (Regberg et al., 2017). A blank sample of an empty sterivex filter was used as a control for low biomass. DNA was examined via Qubit fluorescence and checked via PCR for bacterial signal using full length bacterial primers 8F-1492R (Edwards et al., 1989; Stackebrandt and Liesack, 1993). In the event no DNA was detected via Qubit, it was still positive via PCR. DNA was sent for metagenomic library preparation and sequencing via Illumina HiSeq at the University of Delaware Genomic Sequencing Facility. Raw sequences and MAGs for this project are deposited at NCBI under BioProject PRJNA613490.

### Quality Trim and Assembly

Raw Illumina reads were quality trimmed in CLCBio Workbench version 7.5.1 (Qiagen), with the following parameters: removal of low quality sequence (limit = 0.0016, but rounded to 0.002 by CLCBio, which represents a Phred score of 36 or better); removal of ambiguous nucleotides: no ambiguous nucleotides allowed; removal of terminal nucleotides: 2–12 nucleotides from either end to minimize sequencing errors and enriched 5mers; removal of sequences on length: minimum length 60 nucleotides. Whenever one read of a read pair was excluded due to the quality trim, the entire pair was excluded. Trimmed, paired reads were assembled using IDBA-UD version 1.1.1. with the following settings: –mink 40 –maxk 120 –step 20 –min_contig 300 (Peng et al., 2012). The resulting scaffolds were then used for further genome binning of each reservoir metagenome.

### Phylogeny

The taxonomy of metagenome community members was determined using both Phylosift version 1.0.1 (Darling et al., 2014), with the default parameters, and EMIRGE (Expectation-Maximization Iterative Reconstruction of Genes from the Environment), which is based on the reconstructed 16S rRNA gene sequences from unassembled data (Miller et al., 2011). Contaminants were removed based on comparison to the blank sample and also if the genus was on a list of commonly found kit contaminants (Salter et al., 2014). A maximum likelihood phylogenetic tree of 16S rRNA gene was inferred from the EMIRGE sequences using Mega version 7 using default parameters and 500 bootstrap replicates (Kumar et al., 2016).

### Metagenome-Assembled Genomes (MAGs)

Metagenome assembly of individual samples were subjected to binning using MaxBin version 1.4.2 with the max iteration of 200 (Wu et al., 2014). The taxonomic identity of each resulting MAG was initially determined using Phylosift version 1.0.1 (Darling et al., 2014) with the default parameters. The level of potential contamination and strain heterogeneity in each MAG was evaluated using CheckM 1.0.6 with the “lineage_wf” option (Parks et al., 2015). The VizBin program (Laczny et al., 2015) was then used to visually refine the MAGs to minimize outlier scaffolds. MAGs were then reanalyzed in CheckM for completeness and contamination, keeping only MAGs over 50% complete and less than 10% contaminated, although 2 additional MAGs are reported where those metrics are higher and those should be regarded with caution. Average nucleotide identity (ANI) between the MAGs and reference genomes were calculated using PyANI (Pritchard et al., 2016) implemented in Anvio v5.5 (Eren et al., 2015). Pair-wise average amino acid identity (AAI) was calculated as one-way AAI and two-way AAI using the online tool AAI calculator^1^.

We ran the phylogenomic analysis based on a collection of six ribosomal proteins (Hug et al., 2013) from each MAG that were extracted from the PROKKA annotation (see section below). Also included in the analysis were ribosomal proteins from comparison genomes from closely related microbial groups downloaded from National Center for Biotechnology Information (NCBI). Ribosomal proteins were concatenated and aligned with CLUSTALW in Mega Version 7, and the maximum likelihood tree was also generated with Mega Version 7 using default parameters.

### Functional Annotation

PROKKA version 1.14.6 was used to annotate the metagenomes and MAGs (Seemann, 2014). The presence or absence of functional genes in metabolic pathways was predicted using the BlastKOALA web service provided by the KEGG: Kyoto Encyclopedia of Genes and Genomes website^2^ (Kanehisa et al., 2016).

## Results

### DNA Extraction and Sequence Analysis

DNA was extracted from collected production fluids from five wells, all samples contained water and oil. Low DNA yields were observed, with HA3 being undetectable and other samples having very low DNA quantity: MD1 (0.05 ng/μl), HA6 (0.04 ng/μl), HA2 (4 ng/μl), HA5 (6 ng/μl). All samples did produce a positive reaction with 16S rRNA gene primers while the negative PCR was negative. Given the low DNA concentrations, we processed a blank sample, which was processed alongside the reservoir samples to control for low biomass impacts. We sequenced 5 metagenomes, 1 from each well of the reservoir, in addition to the blank extraction to control for the low biomass anticipated from the DNA extractions. A total of 93 Mbp were sequenced (Table 1).

**TABLE 1:** Metagenome statistics.

	HA2	HA3	HA5	HA6	MD1	Blank
Number of quality trimmed reads	35,209,728	29,490,000	25,746,082	28,359,124	37,840,352	17,888,986
Average length (bp)	134	139	136	151	148	150
Number of assembled contigs	32,312	10,755	21,899	4,104	27,446	8,241
Total base pairs	25,305,506	12,042,019	15,158,776	6,620,593	26,849,201	7,507,877
Average contig size (bp)	783	1,119	692	1,613	978	911
N50 (bp)	723	1,493	587	2,834	2,002	1,056
Largest contig (bp)	178,362	49,149	45,459	36,347	343,822	8,413

### Community Analysis

**FIGURE 1:**
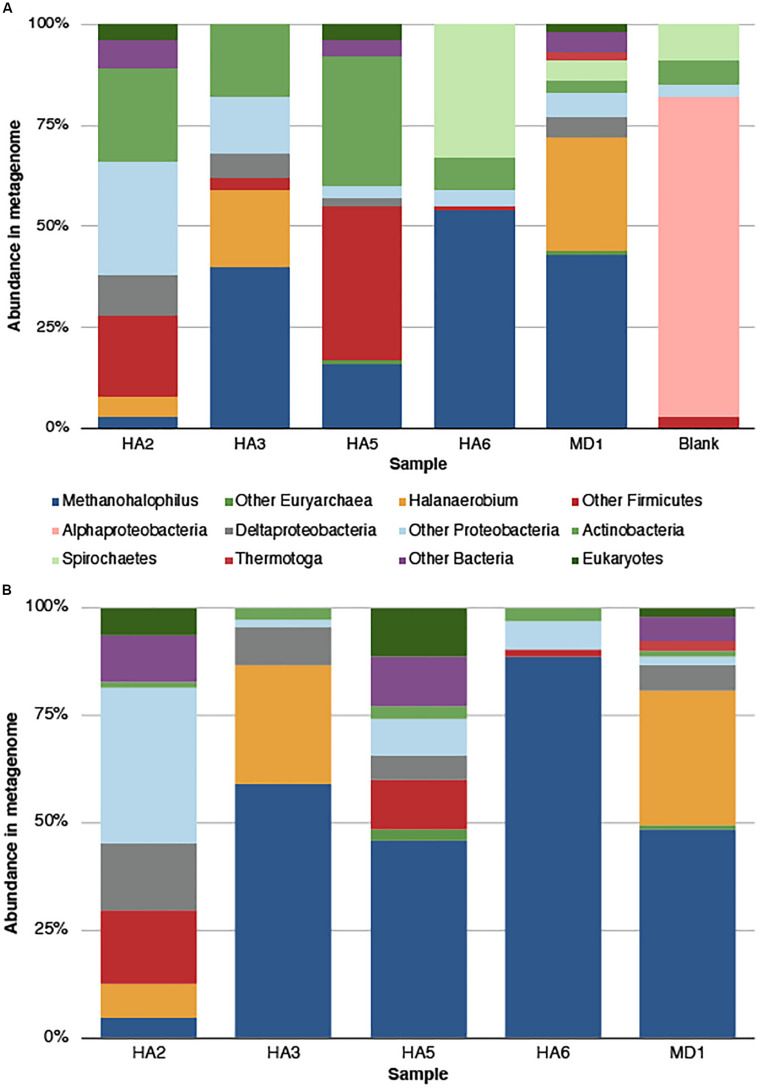
Microbial community composition in the metagenome as determined by Phylosift analysis. **(A)** Quality trimmed data, with all categories determined. The blank sample represents contaminants from the sequencing and extraction process. **(B)** Microbial community composition after contaminants were removed, either by comparison to blank or reference contaminant lists. Categories labeled “Other Bacteria” means that no confident phylogeny was given. “Other Proteobacteria” and “Other Firmicutes” group numerous taxa within those phyla.

Phylosift was used to screen the assembled metagenomes for total microbial populations (Figure 1). Any microbe seen in the blank extraction and also the samples should be discounted, as well as commonly seen contaminants (Salter et al., 2014), including Alphaproteobacteria, Actinobacteria, Spirochetes and “Other Firmicutes” which include *Staphylococcus* and *Streptococcus* (Figure 1A). As such, the most abundant signature across the wells was from *Methanohalophilus* sp., which was most abundant in HA6 (54%) and least abundant in HA2 (3%) (Figure 1B). The second most abundant signature was from *Halanaerobium* sp., with 28% in MD1 and 19% in HA3. It was not found in HA5 or HA6. Other signatures came from other Firmicutes (38% in HA5, 20% in HA2) and Deltaproteobacteria (10% in HA2, not present in HA6). *Thermotoga* were only present in MD1 at 2% relative abundance. A minor fraction of eukaryotic signatures was also seen at 2–4%, and were not detected in the blank extraction.

### Analysis of Metagenome Assembled Genomes

After binning the data into MAGs, we retrieved a majority of *Methanohalophilus* sp. MAGs (Table 2). No bins were generated from HA2. MD1 yielded the highest number of bins, including *Methanohalophilus, Desulfovibrionales, Halanerobium*, and *Bacteroidetes.* The *Bacteroidetes* and *Halanerobium* did not meet the community standards for analysis, as the contamination is over 10%, which may be due to strain heterogeneity. The *Desulfovibrionales* MAG was 100% complete and is in 236 contigs. Considering the *Methanohalophilus* MAGs were the most abundant across samples, we analyzed these as a major focus for this manuscript.

**TABLE 2:** MAG and sequenced relative genome statistics.

Source	HA3	HA5	HA6	MD1	MD1	MD1	MD1	Cultured representatives (database)		
Phylogenetic identity	*Methanohalophilus*	*Methanohalophilus*	*Methanohalophilus*	*Methanohalophilus*	*Desulfovibrionales*	*Halanaerobium*	*Bacteroidetes*	*M. mahii*	*M. halophilus*	*M. euhalobius*
Accession number								NC_014002	NZ_CP017921	GCA_002973515
Bin Size (bp)	1,684,426	1,503,844	1,358,213	1,111,119	3,029,268	4,154,953	4,351,901	2.012,424	2,022,959	1,978,025
Completeness%	82.0%	89.0%	73.5%	58.6%	100.0%	57.8%	96.6%			
Implied Size (bp)	2,054,178	1,689,712	1,847,909	1,896,108	3,029,268	7,163,712	4,533,230			
Contamination% (CheckM)	7.80%	5.60%	1.50%	6.60%	3.60%	16.30%	30.00%			
Strain Heterogeneity	93.30%	44.40%	66.70%	79.00%	0.00%	16.70%	4.60%			
Number of Contigs	348	406	638	370	236	1776	1331	1	1	1
Number of Genes	1,823	1,599	1,436	1,339	2,775	3,935	3,678	1,955	1,987	2,095

The completeness of *Methanohalophilus* MAGs ranged from 58–89%, with implied genome sizes from 1.6–2.1 Mb. The contamination values were below 10% and MAGs had between 348–638 contigs present. Comparing this data to close relatives *M. mahii, M. halophilus*, and *M. euhalobius*, the genome size is similar, as both relatives have 2Mb genomes. Their genomes contain slightly more genes, around 2,000, whereas the MAGs in this study ranged from 1339–1823 genes per MAG (Table 2).

**FIGURE 2:**
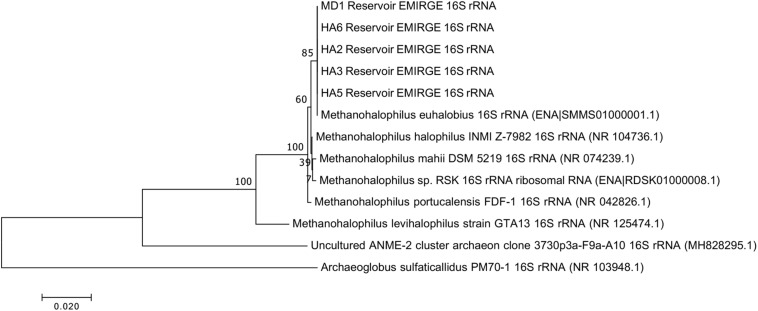
16S rRNA phylogeny based on assembled genes from EMIRGE analysis. The maximum likelihood tree was created in MEGA 7 using default parameters with 500 bootstrap replicates.

**FIGURE 3:**
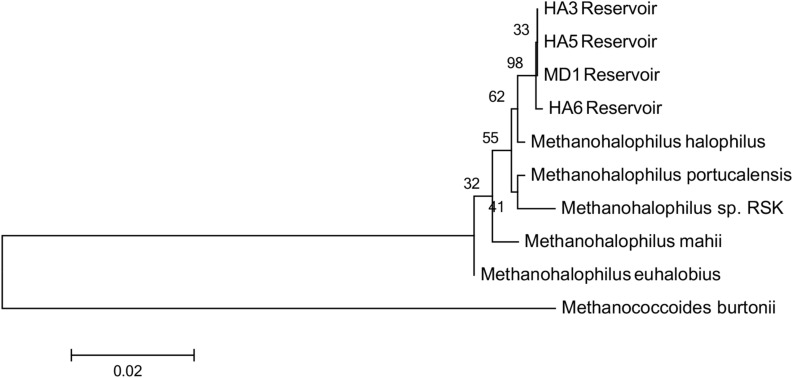
Concatenated ribosomal protein phylogeny of MAGs and close relatives. Ribosomal proteins S4, S10, S11, S12, S13, and L30e were present in all datasets and aligned. The tree is calculated by maximum likelihood in Mega 7 with 500 bootstrap replicates.

**FIGURE 4:**
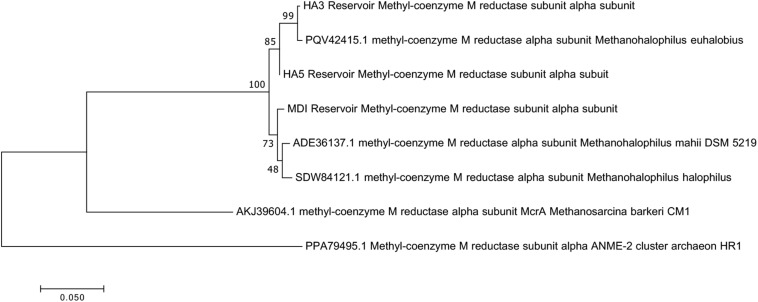
Maximum likelihood phylogeny of *mcrA* genes recovered from MAGs, with 500 bootstrap replicates.

Since not all of the MAGs had discernable 16S rRNA genes, small subunit ribosomal rRNA genes for the *Methanohalophilus* sp. were retrieved from the metagenomes and compared to close relatives (Figure 2). Using shorter sequences (868 bp) from all of the reservoirs, no discernable difference existed between these new sequences and *Methanohalophilus euhalobius*. Using slightly longer sequences (1339–1475 bp) that did not include data from HA2, a slight difference can be seen between HA3, HA6 and the other wells, which matched exactly to *M. euhalobius* (Supplementary Figure 1). To further examine relatedness, we prepared a concatenated ribosomal protein phylogeny using ribosomal proteins (Figure 3). This tree shows that the reservoir signatures are all similar, but in this phylogeny, *M. euhalobius* is not the nearest relative, instead, it is the clade that includes *Methanohalophilus halophilus*. To further examine relatedness, we extracted genes for methyl coenzyme reductase alpha subunit (*mcrA*) from HA3, HA5, and MD1 MAGs. The phylogeny of these functional proteins, which are also utilized as phylogenetic markers for methanogens, shows HA3 being identical, and HA5 being closely related to *M. euhalobius* (Figure 4). The McrA from MD1 is more related to *M. halophilus*.

Considering the incongruent phylogeny of 16S rRNA genes, concatenated ribosomal proteins and McrA sequences, we also examined the average nucleotide identity (ANI) and average amino acid identity (AAI) of the MAGs versus close relatives (Table 3). MAGs were all greater than 97% ANI similarity with each other, although small differences were seen across each MAG, and all MAGs were greater than 97% related to *M. euhalobius*. The MAGs were 91–93% similar to *M. mahii* and *M. halophilus*. The AAI relatedness showed the greatest distance between MAGs from MD1 and HA6 (71%), with the most closely related MAGs being HA3 and HA5 (86%), and all MAGs showing closest relation to *M. halophilus*, ranging from 88–91% AAI.

**TABLE 3:** ANI and AAI values of Methanohalophilus MAG versus isolate genomes.

		*M. mahii*	*M. halophilus*	*M. euhalobius*	HA3	HA5	HA6	MD1
Average nucleotide	*M. mahii*		91.5	91.4	91.4	91.3	91.4	90.7
Identity ANI	*M. halophilus*	91.5		92.6	92.5	92.6	92.8	91.4
	*M. euhalobius*	91.5	92.6		98.3	98.3	98.3	97.6
	HA3	91.5	92.5	98.0		98.5	98.7	97.9
	HA5	91.6	92.6	98.1	98.9		98.8	97.9
	HA6	91.7	92.7	98.0	99.0	98.8		98.1
	MD1	90.9	91.7	97.1	98.0	97.7	97.6	
Average Amino acid	*M. mahii*		87.4	82.8	75.3	73.5	65.8	60.1
identity AAI 1 way	*M. halophilus*	86.6 (92.1)		83.2	74.9	72.9	65.0	59.7
(2 way)	*M. euhalobius*	81.9 (91.6)	81.3 (92.1)		84.1	80.6	71.8	66.2
	HA3	82.6 (90.9)	82.6 (91.8)	90.4 (98.0)		81.7	73.5	68.5
	HA5	84.5 (91.3)	84.5 (91.9)	90.7 (97.6)	86.1 (97.7)		75.1	67.2
	HA6	80.1 (90.8)	79.7 (90.7)	88.0 (95.6)	84.0 (97.0)	80.8 (95.8)		65.8
	MD1	79.9 (87.9)	80.0 (88.5)	88.0 (95.6)	84.5 (95.7)	78.6 (93.9)	71.2 (91.3)	

### Metabolisms Within MAGs and Metagenomes

Across the MAGs and metagenomes, we saw no genes indicative of hydrocarbon usage. The *Desulfovibrionales* MAG suggested that as expected, this microbe performs sulfate reduction. While their genomes are highly contaminated, potentially due to multiple closely related strains, the *Halanaerobium* and *Bacteroidetes* MAGs both appear to live fermentative lifestyles. The metabolic potential of the *Methanohalophilus* sp. MAGs showed that enzymes for methanogenesis from methanol, tri-, di-, and monomethylamines were found across the wells, but no single MAG contained all of the genes, which is likely due to differential completeness of the genomes (Table 4). Searching the metagenome for genes in methylotrophic methanogenesis shows that the majority of wells contain nearly the full pathway. The metabolic potential of the *Methanohalophilus* MAGs did not vary beyond what is known about *M. euhalobius*, so we do presume that genome completion hampered our ability to retrieve metabolic genes for methylotrophic methanogenesis, but that these are methylotrophic methanogens as this is highly conserved in the *Methanohalophilus* lineage (Guan et al., 2019).

**TABLE 4:** Methyltransferase genes for methanogenesis.

*Methanohalophilus* MAGs	HA3	HA5	HA6		MD1
Methanol (*mtaA*)	X	X	X		X
methylamine-specific corrinoid protein (*mtbA*)	X	X	–		–
Monomethylamine methyltransferase (*mtmB*)	–	X	X		–
Dimethylamine methyltransferase (*mtbB*)	X	X	X		–
Trimethylamine methyltransferase (*mttB*)	–	–	–		–

**Metagenomes^1^**	**HA2**	**HA3**	**HA5**	**HA6**	**MD1**

Methanol (*mtaA*)	–	X	X	X	X
methylamine-specific corrinoid protein (*mtbA*)	X	X	X	X	X
Monomethylamine methyltransferase (*mtmB*)	–	X	X	X	X
Dimethylamine methyltransferase (*mtbB*)	–	X	X	X	X
Trimethylamine methyltransferase (*mttB*)	–	X	–	X	–

*^1^Based on BLASTP searches of the nr database, the methyltransferase sequences from metagenomes were all from various Methanohalophilus species, including M. euhalobius, M. halophilus, and M. portucalensis.*

Since the *Methanohalophilus* genus is well known for the production of glycine betaine (GB), used as a compatible solute to allow salt tolerance (Guan et al., 2019), and GB can be fermented by *Halanaerobium* spp., producing trimethylamine, which in turn can feed *Methanohalophilus* spp. (Daly et al., 2016), we examined our data for genes that could indicate GB fermentation. The single MAG from MD1 of *Halanaerobium* contained all genes needed for GB fermentation (Table 5). We then examined all metagenomes for genes in the large glycine reductase family, as this is the family that includes GB reductase. Using a BLAST comparison with the GB reductase gene (noted by the GSFMP active site; Daly et al., 2016), HA3 and MD1, both samples that contained *Halanaerobium*, produced significant matches, with homology over 75% and expectancy values under 1e^–50^. A glycine reductase gene was found in HA3, however, it contained the GNCVS active site, which is not specific for GB. Only MD1 contained a complete set of subunits for GB reductase with the GB-specific GSFMP active site. When these genes were analyzed by BLAST, the top hits were all to *Halanaerobium* species. Considering the high strain heterogeneity in the MAG from this well, we interpret this to mean there are multiple GB-fermenting *Halanaerobium* species in MD1.

**TABLE 5:** Glycine betaine reductase genes.

Halanaerobium MAGs	MD1	–		
betaine reductase complex component A	X	–	–	–
betaine reductase complex component B subunit alpha^1^	X	–	–	–
betaine reductase complex component B subunit beta	X	–	–	–
betaine reductase complex component C subunit alpha	X	–	–	–
betaine reductase complex component C subunit beta	X	–	–	–

**Metagenomes**	**HA3**	**HA5**	**HA6**	**MD1**

betaine reductase complex component A	X	–	–	X
betaine reductase complex component B subunit alpha	X	–	–	X
subunit alpha active site^2^	GNCVS	–	–	GSFMP
betaine reductase complex component B subunit beta	X	–	–	X
betaine reductase complex component C subunit alpha	–	–	–	X
betaine reductase complex component C subunit beta	X	X	–	X

*^1^Betaine reductase complex B distinguishes this enzyme from others in its family including glycine reductase, sarcosine reductase and proline reductase. ^2^Active sites determine specificity of the glycine reductase. GSFMP is glycine betaine-specific. GNCVS is a generic glycine reductase.*

## Discussion

The low DNA yield of these samples increased the potential for contamination in the sequencing process. However, preparing a blank sample that followed the process of extraction, library preparation and sequencing allowed the ability to confidently determine which taxa to discard as laboratory contaminants, as well as considering those established as common contaminants. Disregarding these contaminant taxa, we see that these oil wells have limited diversity in regards to phyla that are present, consisting of *Methanohalophilus*, *Halanaerobium* and smaller contributions from Firmicutes, Deltaproteobacteria and *Thermotoga*.

The most abundant inhabitant of these oil wells is the methanogenic lineage of *Methanohalophilus.* Detailed phylogenetic analysis of metagenome-assembled genomes shows that this methanogen is a close relative of previously described halophile, *Methanohalophilus euhalobius*, also isolated from an oil reservoir system. While the previously held theory that halophilic environments favor methylotrophic methanogens due to energetic constraints (Sorokin et al., 2017), this system presents more potential influences on methanogenic lineages than energy alone. Mainly, the addition of methanol to the wells to prevent methane hydrate formation may have been a driving force behind the growth of methylotrophic methanogens.

Another influence on the growth of methylotrophic methanogens could the presence of *Halanaerobium* sp., also detected in the metagenomic analysis. It was previously shown that the fermentation of GB to trimethylamine by *Halanaerobium* furthered the growth of *Methanohalophilus* in fractured shale production water (Daly et al., 2016). In our study, however, the species abundances of *Halanaerobium* and *Methanohalophilus* did not match, for example, in HA5 and HA6, no *Halanaerobium* is seen, yet there is an abundance of *Methanohalophilus* signal. Therefore, while we found evidence that GB fermentation is possible in some of these wells, we conclude that the industrial methanol addition may be a greater factor in the dominance of *Methanohalophilus* in these wells. Since no additional hydrocarbon genes were found, the sulfate reducer and other fermentative organisms may be processing biomass or organic acids found in the environment. A full metabolomic profile, along with more detailed genomics, would be needed to reconstruct complete pathways of metabolic interdependencies.

We have not yet examined detailed evolutionary processes that may be occurring subsurface in these wells. However, we do note that although these wells are connecting to the same production interval, additional compartmentalization of the production zones is likely, further supported by the fact that individual wells had differentiated microbial populations and that the methanogens within these systems were not identical. We suspect that these organisms may be infrastructure contaminants, selected due to their tolerance of high salinity and their growth on industrial additions *in situ*. This study suggests that some of the broadly observed lineages in both conventional and unconventional (hydraulically fractured) reservoirs may be allochthonously introduced through industrial development. While indigenous communities and organisms exist in these deep environments, care must be taken in the interpretation and study of subsurface hydrocarbon reservoir communities.

## Data Availability Statement

The datasets presented in this study can be found in online repositories. The names of the repository/repositories and accession number(s) can be found below: https://www.ncbi.nlm.nih.gov/genbank/, PRJNA613490.

## Author Contributions

ZS provided the samples. RL-Z performed laboratory work. GC, RL-Z, and JB analyzed the data. All authors contributed to the writing of the manuscript.

## Conflict of Interest

ZS was employed by ExxonMobil Research & Engineering. The authors declare that this study received funding from ExxonMobil Research and Engineering and ExxonMobil Upstream Research Company. The funders had the following involvement in the study: Sample collection.

## Footnotes

1 http://enve-omics.ce.gatech.edu/aai/

2 http://www.kegg.jp/blastkoala/
