# Supplementary material for: Methanogens Within a High Salinity Oil Reservoir From the Gulf of Mexico
Source: Front Microbiol. 2020 Sep 18;11:570714. doi: 10.3389/fmicb.2020.570714 (PMC7530209; doi:10.3389/fmicb.2020.570714)
Supplement: Supplementary file 1 [file Image_1.pdf]

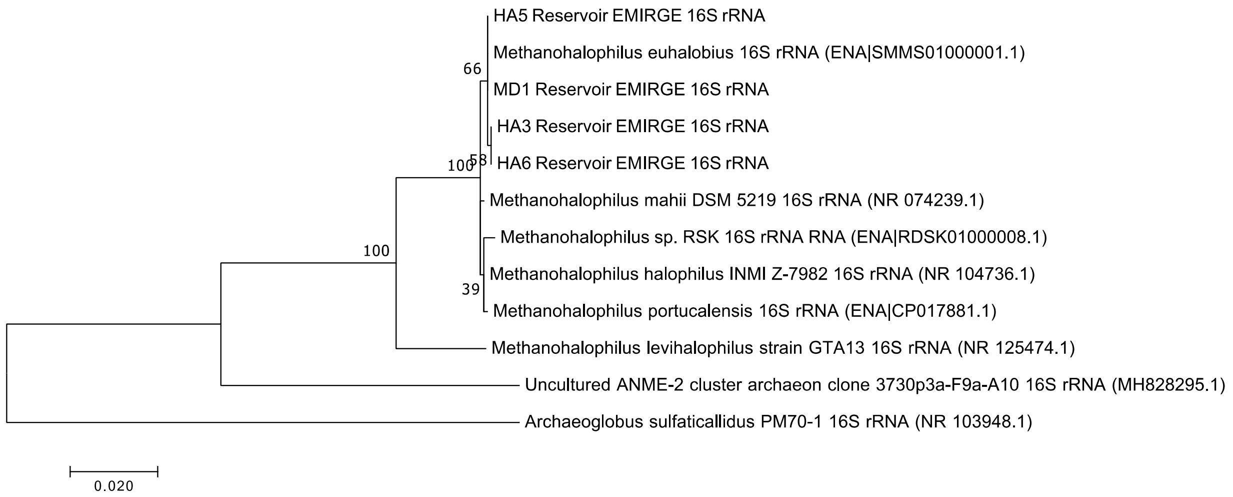

Supplementary Figure 1: 16S rRNA gene phylogeny based on EMIRGE reconstruction. Only long sequences are used in this maximum likelihood tree, so not every reservoir is represented. The tree was created in MEGA 7 using default parameters., with 500 bootstrap replicates.
